# Safety and Efficacy of Very Low‐Dose Multi‐Nut Oral Immunotherapy in Children

**DOI:** 10.1002/clt2.70125

**Published:** 2025-12-12

**Authors:** Julia E. M. Upton, Carmen H. Li, Alireza Berenjy, Alana Galper, Xiaojun Yin, Alper Celik, Lucy Duan, Samantha Wong, Christina M. Ditlof, Kristen E. San Diego, Jennifer A. Hoang, Moshe Ben‐shoshan, Akash Kothari, Lisa Hung, Mikhail Monteiro, Wut Hmone Phue, Theo J. Moraes, Thomas Eiwegger

**Affiliations:** ^1^ Division of Immunology and Allergy, Food Allergy and Anaphylaxis Program The Hospital for Sick Children Toronto Ontario Canada; ^2^ Department of Pediatrics University of Toronto Toronto Ontario Canada; ^3^ Translational Medicine Program Research Institute Hospital for Sick Children Toronto Ontario Canada; ^4^ Institute of Medical Science University of Toronto Toronto Ontario Canada; ^5^ Centre for Computational Medicine Research Institute Hospital for Sick Children Toronto Ontario Canada; ^6^ Department of Immunology University of Toronto Toronto Ontario Canada; ^7^ Department of Pediatrics, Division of Allergy and Clinical Immunology Montreal Children's Hospital Montreal Ontario Canada; ^8^ Karl Landsteiner University of Health Sciences Krems an der Donau Austria; ^9^ Department of Pediatric and Adolescent Medicine University Hospital St. Pölten Sankt Pölten Austria

**Keywords:** food allergy, immunotherapy clinical, oral immunotherapy, pediatric

## Abstract

**Background:**

Oral immunotherapy (OIT) is a management strategy for food allergies, typically one at a time, with maintenance doses ≥ 300 mg protein. However, 30% of allergic children have multiple trigger foods, and large maintenance doses are associated with side effects. If efficacious, Very Low‐Dose OIT (VLOIT) may enhance safety in multi‐OIT.

**Methods:**

Eighteen children with allergies to 2–5 nuts (tree nuts, peanuts) were enrolled (NCT03799328). Oral food challenge (OFC)‐confirmed allergies to their nut mix at ≤ 444 mg protein each nut followed by initiation of an open‐label mix of 4 mg protein/nut with dose increases every 2 months up to a maintenance dose of 30 mg protein/nut. After 18 months, an exit‐OFC assessed allergic threshold changes, with a maximum of 2040 mg protein/nut. Efficacy was evaluated using pre‐post treatment and proportional analyses (Wilcoxon signed‐ranks, two‐tailed Fisher's test).

**Results:**

The median age at enrollment was 5.0 years (IQR 3.13–9.62). The baseline median tolerated dose was 10 mg protein/nut (IQR 3–100 mg). Three withdrew, one did not reach the target maintenance but was invited for the exit OFC, resulting in 15/18 eligible for exit OFC. The median tolerated dose at exit OFC was 1000 mg (IQR 300–1000 mg), with a significant difference from baseline (*p* < 0.0001). Ten out of 15 participants tolerated the maximum dose (*p* < 0.0001). Intention‐to‐treat analysis showed that 14/18 children met pre‐defined efficacy measures: tolerated 5X their baseline dose or ≥ 300 mg (*p* < 0.001). No patients required epinephrine during treatment.

**Conclusions:**

VLOIT led to a significant increase in the tolerated dose to multiple nuts.

**Abbreviations**
AE: Adverse Event
EI: Emotional Impact
FAQLQ: Food Allergy Quality of Life Questionnaire
FRA: Food Related Anxiety
IgE: Immunoglobulin E
IgG4: Immunoglobulin G4
IQR: Interquartile Range
OFC: Oral Food Challenge
OIT: Oral Immunotherapy
SAE: Serious Adverse Event
SD: Standard Deviation
SDR: Social and Dietary Restrictions
SPT: Skin Prick Test
TAE: Treatment Associated Adverse Event
VLOIT: Very Low‐Dose Multi‐Oral Immunotherapy

## 1 Introduction

Food allergies, primarily peanut and tree nut allergies, affect up to 8% of children in North America [1]. Oral immunotherapy (OIT) treats food allergies, typically one at a time, and is now offered as a management strategy for food allergies in clinical practice in Canada and the United States by some Allergist‐Immunologists and by the European Society of Allergy and Clinical Immunology [2, 3, 4]. However, many children have multiple allergies, and the large maintenance doses typically used in OIT are associated with increased risk of side effects, including anaphylaxis, relative to OIT with lower maintenance doses, which necessitates many clinic visits for supervised dose‐escalation.

Multi‐allergen OIT, treating multiple food allergens concurrently, has been successfully conducted using large maintenance doses up to 4000 mg protein per allergen in combination with the biologic omalizumab [5]. Low doses have the potential to enable more accessible multi‐allergen OIT dosing without adjuvants. Low doses may be more easily tolerated and, in the context of extended treatment duration, may positively contribute to the success rate of immunotherapy [6, 7].

Currently, the lower limit of OIT treatment necessary to provide protection has not been determined. However, the amount of food a patient tolerates after allergen immunotherapy is proportionally much higher than the maintenance dose, even for very small doses. One of the smallest reported target maintenance doses for OIT was 75 mg of walnut protein, which was well tolerated by three children who successfully completed an Oral Food Challenge (OFC) with 450 mg of walnut protein [8] Thus, a very low target maintenance dose may still provide meaningful protection against accidental exposure.

Very Low‐Dose Multi‐OIT (VLOIT) (NCT03799328) is a single‐arm, open‐label study of the intervention of low‐dose multiple‐nut OIT in tree nut and peanut‐allergic children. This trial integrates two unmet needs of OIT: first, to study the efficacy of low maintenance doses, which may be more feasible for wide practice due to safety and reduced visits, and second, to simultaneously safely treat multiple food allergies. Our primary outcome is desensitization to the allergic foods as assessed by change in maximum tolerated dose by oral food challenge (OFC) and whether the participant could consume at least 5 times the baseline tolerated dose 18 months after initiating VLOIT.

## 2 Methods

### 2.1 Ethics Approval

The Very Low‐Dose Multi‐OIT (VLOIT) was reviewed by the Research Ethics Board at The Hospital for Sick Children (#1000060633) and is registered as a clinical trial (NCT03799328). Informed consent was obtained prior to screening.

### 2.2 Study Population

The study was initiated in May 2019, and its primary visit completion was concluded in November 2022. Children aged 6 months to 15 years with allergies to 2–5 tree nuts and/or peanuts (nuts) were recruited. Inclusion criteria included allergic status confirmed by positive OFC to a nut mix at ≤ 00 mg protein/nut (≤ 444 mg protein/nut cumulative), serum immunoglobulin E (IgE) > 0.35 kilounits/L (kU/L) (determined by UniCAP within the past 12 months) and/or a SPT to nut ≥ 3 mm diameter compared to control (saline).

Exclusion criteria were defined by a history of frequent or repeated severe episodes of anaphylaxis, treatment with omalizumab, use of other non‐traditional forms of allergen immunotherapy or biologic therapy in the prior 12 months, history of eosinophilic gastrointestinal disease, uncontrolled asthma [9], the use of oral beta blockers and/or angiotensin‐converting enzyme inhibitors. Other exclusion criteria included failure to tolerate 4 mg protein/nut during the baseline OFC or during the first desensitization day (Supporting Information S1: Table S1), which is a comparable minimum safety threshold reported in other OIT studies [10, 11] and we aimed to have a typical population to study very low doses, not just children with very low thresholds of reaction.

### 2.3 Trial Design

**FIGURE 1:**
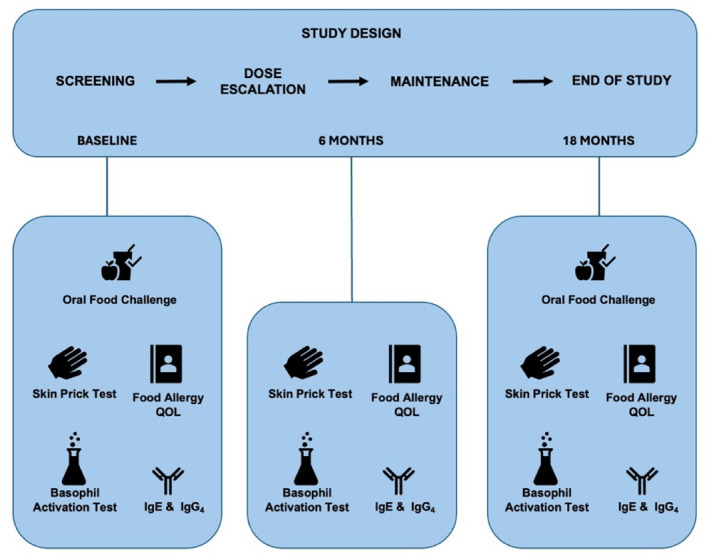
Study design overview. Participants allergic to 2–5 nuts underwent an oral food challenge (OFC) at screening, followed by bi‐monthly visits for dose escalation to 30 mg protein/allergen. Once participants achieved 30 mg protein/allergen, they remained on 30 mg protein/allergen maintenance dosing for approximately 12 months or until the 18‐month time point. Participants then underwent a second OFC at the exit visit. Blood sample collection, skin prick testing, and Quality of Life Questionnaires are conducted at baseline when 30 mg protein/nut dosing was achieved, and at the 18‐month end of the study visit.

VLOIT was prospectively conducted based on the child's allergic status (peanut, walnut, hazelnut, almond, cashew, pistachio, pecan, macadamia) with the target maintenance about 30 mg each nut protein/day. After OFC, children were initiated on an open‐label protein‐calibrated mix of approximately 4 mg of each nut protein, with medically supervised dose‐escalation every 2 months (Supporting Information S1: Table S2). The allergen was administered via store‐bought nut butter measured with micro‐spoons. Eighteen months after the start of multi‐nut VLOIT, a repeat semi‐logarithmic dosed OFC (exit OFC) to a cumulative maximum of 2040 mg protein/nut was completed to assess the change in allergic threshold and clinical response. Immunological characterization, including SPT and antibody titers, were conducted at baseline, after the dose escalation (approximately 6 months) and after the completion of the study (18 months) (Figure 1).

### 2.4 Primary Outcome

The primary outcome was the change in reaction threshold and tolerated dose before and after treatment at 18 months as assessed by open OFC. The proportion of subjects who achieved desensitization as determined by tolerating specified challenge doses of nut protein with no more than mild symptoms was calculated. The specified primary outcome was reaching a dose that is at least 5 times higher than the baseline tolerated dose (or tolerating a cumulative dose of 2040 mg protein/nut food challenge). The primary immunological parameter assessed was the change in allergen‐specific IgG4 from baseline, to post‐escalation (approximately 6 months), and at the end of OIT (18 months). Key secondary outcome measures included whether participants could tolerate 300 mg protein/nut (444 mg/nut cumulative). Mathematical modeling indicates that very few allergic reactions would occur due to accidental exposures to nuts while tolerating 300 mg protein [12]. We also assessed desensitization as the change in the amount tolerated at baseline OFC versus the amount tolerated at 18 months.

### 2.5 Oral Food Challenge Protocol

All oral food challenges (OFC) and desensitization procedures were done with commercially available peanut/tree nut protein sources in a hospital‐based clinic. The foods for OIT were combined and eaten as one dose as outlined in prior multi‐OIT [13]. The nut mix was calculated using the protein quantification from the nutrition information and was then mixed in a food vehicle, such as pudding or fruit puree. Doses were given by the study nurse or physician every 15–20 min. Initial OFC was to up to 444 mg (started at 1 mg and ended at 300 mg). Exit OFC was up to 2040 mg (started at 10 mg and ended at 1000 mg (Supporting Information S1: Table S3)). When the participant demonstrated objective signs of an allergic reaction, the challenge was stopped and the reaction was treated. Participants were kept under observation for at least 2 hours after OFC. Given the similarity of allergens between walnuts and pecans, only walnut was used in the trial. Both cashew and pistachio were used.

### 2.6 Dose Escalation Protocol

Dose escalation steps are outlined in Supporting Information S1: Table S4. Participants were observed for at least 1 hour in keeping with clinical practice [7]. If the participant did not tolerate up‐dosing, the timeline was extended, and dose escalation was reattempted at a follow‐up visit.

### 2.7 Formulation of OIT Product and Home Dosing Protocol

Individualized protein‐calibrated nut butter mixes were prepared for each participant for OFC and home dosing. Pure nut butters contain about 6–7 g (6000–7000 mg) of nut protein per 28 g, equivalent to roughly 1000 mg per teaspoon. The research staff weighed a custom mix (e.g., walnut and peanut butters for a walnut‐peanut OIT participant) with equivalent amounts of each nut protein per unit volume as dictated by the specific nut butter's nutritional label information. Participants were given mini‐measuring spoons measuring down to 1/64 tsp. Dosing accuracy was taught during the research unit on the initial desensitization day, using the bulk nut butter mixture created for them for use at home. A fresh mixture of their individual nut mix was provided to participants after each dose escalation visit to continue home dosing with mini‐spoons.

### 2.8 Mechanistic Studies

The SPT and the measurement of allergen‐specific IgE and IgG4 are described in the supplementary information.

### 2.9 Exploratory Studies

The Food Allergy Quality of Life Questionnaires (FAQLQ‐PF, CF, and TF) were conducted to assess the health‐related QoL of parents and participants. The validated FAQLQs were conducted based on the patient's age, and both parent forms (PF) and child forms (CF) were available (FAQLQ‐PF 0–12 years; FAQLQ‐CF 8–12 years; FAQLQ‐TF 13–17 years).

### 2.10 Adverse Events

Participants were provided with a diary to record urticaria, wheezing, oropharyngeal symptoms, and administration of any medication, including antihistamines, albuterol, or epinephrine. An adverse event (AE) was defined as the following: (1) A new event which was not pre‐existing at the initial study drug administration, (2) A pre‐existing event recurs with increased intensity or frequency after study drug administration, (3) An event present at the time of study drug administration exacerbated following the initial study drug administration. A Serious Adverse Event (SAE) was defined as Grade 3 (severe) anaphylaxis on the Brown scale [5]. Reactions during OFC were considered expected.

### 2.11 Analysis

The sample size was determined by a pre‐study power calculation described in the supplementary section. We computed averages, medians, and proportions for demographic and immunological measurements, comparing baseline and post‐OIT values as appropriate. Paired non‐parametric test statistics, including Friedman's test and Dunn's multiple comparisons, and Wilcoxon signed‐rank tests, were conducted. The proportion of patients who met the 5 times baseline OFC threshold or who tolerated the ≥ 300 mg protein/nut dose was evaluated using a two‐tailed Fisher's exact test. Analyses were performed with GraphPad Prism 10 for Mac (La Jolla, CA) or Stata/SE 13.1 (College Station, TX). Test results *p* < 0.05 were considered significant.

## 3 Results

### 3.1 Study Participant Enrollment and Withdrawal

**FIGURE 2:**
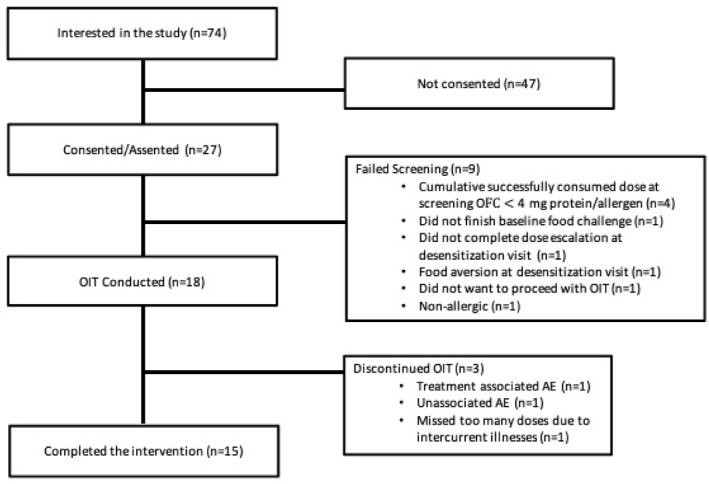
Consort diagram for very low‐dose multi‐oral immunotherapy. Twenty‐seven patients consented to the study. Nine patients did not meet the inclusion criteria and did not proceed to intervention. Of 18 patients, 3 withdrew from the study, 14 completed the 30 mg protein/nut up dose target, and 1 achieved 16 mg protein/nut up dose. Fifteen patients completed the 18‐month end of maintenance timepoint. AE: Adverse Event.

Twenty‐seven children were consented and screened, and 18 participants were enrolled. Study progression is shown in Figure 2. Baseline demographics are shown in Table 1, and baseline clinical characteristics are shown in Supporting Information S1: Table S5. One participant received OIT for each of the following combinations: (walnut, pistachio, cashew); (pistachio, cashew); (pistachio, cashew, macadamia); and (hazelnut, peanut). Four participants received OIT for walnut, pistachio, cashew, and hazelnut. Four participants received OIT for walnut, pistachio, cashew, hazelnut, and peanut. Three participants received (walnut, peanut) and (pistachio, cashew, peanut) OIT, respectively. The median age at enrollment was 5.0 years (IQR 3.13–9.62), and 12/18 (66.7%) were male. Three participants withdrew from the study: one missed too many OIT doses due to illness, one experienced mild OIT‐associated gastrointestinal symptoms, and one experienced an AE unassociated with OIT (ulcerative colitis). The latter participant was assessed by Gastroenterology at The Hospital for Sick Children for eosinophilic esophagitis through an upper endoscopy, which was negative.

**TABLE 1:** Very low‐dose multi‐OIT patient demographics.

Participants *N* (%)	Participants who received VLOIT (%)
Total	18
Males	12 (66.7)
Median age at start of OIT (IQR)	5.0 (IQR 3.13–9.62)
Atopic history (%)	
Asthma	6 (33.3)
Atopic dermatitis	5 (27.8)
Allergic rhinitis	12 (66.7)
Peanut and/or tree nut allergy (%)	
Walnut	12 (66.7)
Pistachio	14 (77.8)
Cashew	14 (77.8)
Hazelnut	9 (50.0)
Peanut	11 (61.1)
Macadamia	1 (5.6)
Almond	0 (0.0)

### 3.2 OIT Clinical Outcomes

#### 3.2.1 Very Low‐Dose Multi‐OIT Protocol Adherence

The median time to achieve 30 mg protein/nut maintenance dose from enrollment was 304.50 days (IQR 280.75–339.75). Reduced clinic capacity and patient fears of hospital visits during the 2020–2022 years of the COVID‐19 pandemic account for the extended time to achieve maintenance. The median time from OIT initiation to exit OFC, planned for 18 months (548 days), was very close to target at 564.00 days (IQR 556.00–590.00). An estimate of treatment dosing adherence, based on the number of reported missed doses and the total treatment days, was 99.6%.

#### 3.2.2 Increase in Cumulative Tolerated Dose

**FIGURE 3:**
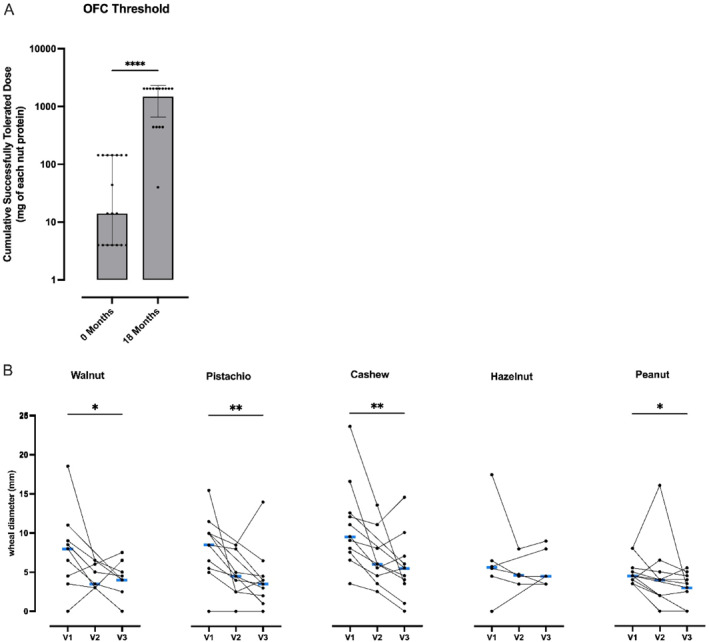
Increase in the cumulative tolerated dose of allergen protein and reduction of skin prick tests over 18 months. (A) The cumulative amount of protein per allergen consumed at baseline and at the end of the maintenance timepoint at 18 months (median and IQR). Fifteen patients underwent 18 months and 14 completed the treatment protocol to 30 mg dosing. Wilcoxon signed ranks test. ****(*p* < 0.0001) (B) Allergen‐specific wheal diameter at baseline (V1), post‐dose escalation visit (V2), and end of maintenance exit visit at 18 months (V3). Positive SPT is defined as > 3 mm diameter compared to negative (saline) skin test. Median is indicated in blue. Friedman and Dunn's test for multiple paired values of three time points. Walnut (*n* = 7, Friedman test N.S.), Pistachio (*n* = 9, Friedman test *p* < 0.01, Dunn's test V1 vs. V3 *p* < 0.05), Cashew (*n* = 9, Friedman *p* < 0.05, Dunn's test V1 vs. V2 *p* < 0.05), Hazelnut (*n* = 4, Friedman test *p* < 0.05, Dunn's test N.S.), Peanut (*n* = 10, Friedman test *p* < 0.05, Dunn's test V1 vs. V3 < 0.05). Wilcoxon signed‐rank test for paired values at baseline (V1) and end of maintenance period (V3) (plotted). Walnut (*n* = 9 *p* < 0.05), Pistachio (*n* = 11 *p* < 0.01), Cashew (*n* = 11 *p* < 0.01), Hazelnut (*n* = 6 N.S.), Peanut (*n* = 11 *p* < 0.05). N.S.: Non‐significant, SPT: Skin prick test *(*p* < 0.05), **(*p* < 0.01).

The median tolerated dose at baseline challenge was 10 mg protein/nut (IQR 3–100 mg). The median tolerated dose at exit OFC for treated participants was 1000 mg protein/nut (IQR 300–1000), with 10/15 tolerating the 1000 mg dose and therefore the cumulative maximum dose of 2040 mg protein/nut. One participant did not reach the target maintenance, achieving approximately 16 mg protein/nut, but was invited for the exit OFC. Thus, 15/18 were eligible for the exit OFC. The comparison of the tolerated dose at OFC to baseline showed a highly significant increase (*p* < 0.0001; Wilcoxon signed‐ranks test; Figure 3). Additionally, of the 15 participants invited to the exit OFC, 10 experienced a decrease in OFC reaction severity, 0 (IQR 0, 2) compared to baseline. 2 (IQR 1,3 *p*‐value: 0.0078 (Supporting Information S1: Table S6).

If we consider only those 14 children who achieved the 30 mg maintenance dosing (per protocol), 13/14 (93%) tolerated least 5 times their baseline eliciting dose (or the maximum dose) and tolerated the ≥ 300 mg protein/nut dose (*p* < 0.001 vs. baseline) and 10/14 (71%) tolerated the maximum amount (1000 mg dose, 2040 mg cumulative), (*p* < 0.001 vs. baseline).

From the original 18 children, including withdrawals, 14/18 (78%) children tolerated at least 5 times their baseline eliciting dose (or the maximum dose) and tolerated the ≥ 300 mg protein/nut dose (*p* < 0.001 vs. baseline). The participant who achieved only 16 mg protein/nut maintenance successfully consumed 300 mg protein (cumulative 440 mg protein/nut) at 18 months, which was 100 times greater than their baseline tolerated dose (3 mg protein/nut, cumulative 4 mg protein/nut).

#### 3.2.3 Very Low‐Dose Multi‐OIT Suppresses IgE and Induces IgG4 Blocking Antibodies to Albumin Proteins

Patients receiving multi‐OIT demonstrated multi‐desensitization to all the nuts in the prescribed nut mix. This was reflected in a significant decrease in SPT diameter for all nuts between baseline and 18 months (walnut *p* < 0.05, pistachio *p* < 0.01, cashew *p* < 0.01, peanut *p* < 0.05) except for hazelnut, which followed a similar trend, but had a smaller sample size (Figure 3).

**FIGURE 4:**
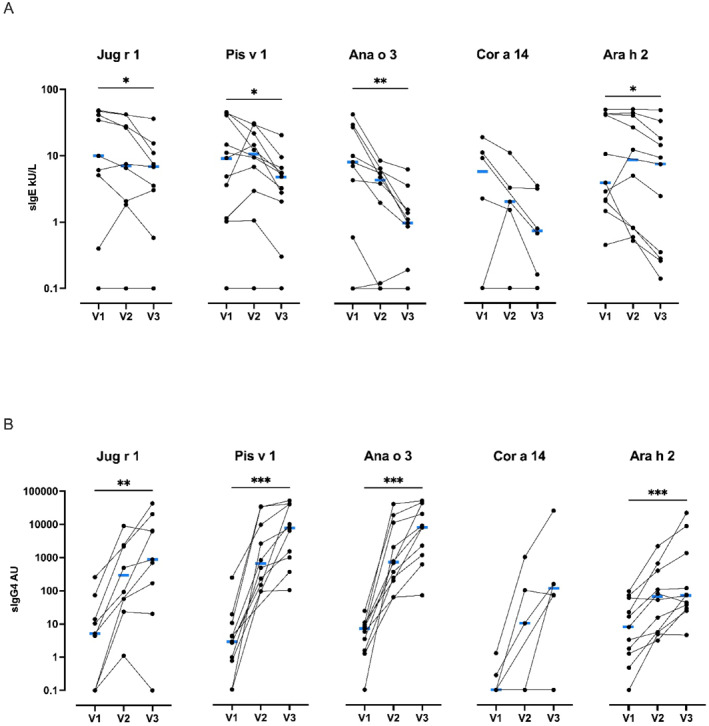
Allergen‐specific IgE and IgG4 change during Very Low‐Dose Multi‐OIT. (A) Allergen‐specific IgE at baseline (V1), post dose‐escalation visit (V2), and end of maintenance exit visit at 18 months (V3). Friedman and Dunn's test for multiple comparisons of paired values at all three time points and end of maintenance exit visit at 18 months. Median is indicated in blue. Walnut Jug r 1 (*n* = 8, Friedman N.S.), Pistachio Pis v 1 (*n* = 10, Friedman test *p* < 0.05, Dunn's test V2 vs. V3 *p* < 0.05), Cashew Ana o 3 (*n* = 10, Friedman test *p* < 0.01, Dunn's test V1 vs. V3 *p* < 0.01), Hazelnut Cor a 14 (*n* = 5, Friedman test N.S.), Peanut Ara h 2 (*n* = 10, Friedman test *p* < 0.01, Dunn's test V1 vs. V3 *p* < 0.01, V2 vs. V3 *p* < 0.01)Wilcoxon signed‐rank test for paired values baseline (V1) and end of maintenance (V3) visits (plotted). Walnut Jug r 1 (*n* = 9, *p* < 0.05), Pistachio Pis v 1 (*n* = 11 *p* < 0.05), Cashew Ana o 3 (*n* = 11 *p* < 0.01), Hazelnut Cor a 14 (*n* = 6, N.S.), Peanut Ara h 2 (*n* = 11 *p* < 0.05). (B) Allergen‐specific IgG4 at Baseline (V1), post‐escalation visit (V2) and end of maintenance exit visit at 18 months (V3). Friedman and Dunn's test for multiple comparisons of paired values at 3 time points. Median is indicated in blue. Walnut Jug r 1(*n* = 8, Friedman test *p* < 0.01, Dunn's test V1 vs. V2 *p* < 0.05, V1 vs. V3 months *p* < 0.01), Pistachio Pis v 1 (*n* = 10, Friedman test *p* < 0.0001, Dunn's test V1 vs. V3 *p* < 0.0001), Cashew Ana o 3 (*n* = 10, Friedman test *p* < 0.0001, Dunn's test V1 vs. V3 *p* < 0.0001), Hazelnut Cor a 14 (*n* = 5, Friedman test N.S.), Peanut Ara h 2 (*n* = 10, Friedman test *p* < 0.001, Dunn's test V1 vs. V3 *p* < 0.001). Wilcoxon signed‐rank test for paired values baseline (V1) and end of maintenance (V3) (plotted). Walnut Jug r 1 (*n* = 9 *p* < 0.01), Pistachio Pis v 1 (*n* = 11 *p* < 0.001), Cashew Ana o 3 (*n* = 11 (*p* < 0.001), Hazelnut Cor a 14 (*n* = 6, N.S.), Peanut Ara h 2 (*n* = 11 *p* < 0.001). N.S.: Not Significant, *(*p* < 0.05) ** (*p* < 0.01) ***(*p* < 0.001).

In addition, a statistically significant reduction of allergen‐specific IgE and the induction of allergen‐specific IgG4 blocking antibodies to 2S albumins of the treatment nuts was significantly increased between baseline and 18 months except for hazelnut, likely due to sample size (Figure 4).

#### 3.2.4 Very Low‐Dose Multi‐OIT Was Overall Safe and Well‐Tolerated

There were no treatment‐related, protocol‐defined severe adverse events (AE), hospitalizations, or deaths. No participants required epinephrine for home reactions or dose‐escalation visits (Table 2). Three of the 18 enrolled patients experienced no treatment‐associated adverse reactions during home‐dosing or the dose escalation visits. One patient experienced one mild AE during a dose‐escalation visit (itch, sneezing) and no other treatment‐associated AEs.

**TABLE 2:** Adverse events during home‐dosing in very low‐dose multi‐OIT.

Daily dosing associated adverse events during dose escalation & maintenance phase	
Total number of OIT daily doses with accompanying AE	# Patients who experienced AEs/# enrolled patients
641	14/18

Symptom categories		# OIT doses with symptoms	# Patients affected	# Doses antihistamine for symptoms	# Patients who received temporary dose adjustment following symptoms
Dermatological	General skin itch	35	6	0	0
	Eczema flare	2	1	0	0
	Hives, urticaria, rash	17	5	1	1
Local oral reaction	Itch of lips, mouth, throat, tongue	241	10	1	2
	Hive on lips, mouth	4	4	0	0
	Redness, swelling of lips, mouth	15	4	0	0
	Pain or discomfort of lips, mouth, throat, tongue	16	5	0	0
Gastrointestinal	Nausea	14	3	1	1
	Vomiting	4	3	2	1
	Diarrhea	0	0	0	0
	Abdominal pain	348^a^	7	3	2
	Acid reflux	7	1	0	0
Respiratory	Cough w/o dyspnea^b^	2	2	0	0
	Wheezing/Asthma exacerbation	0	0	0	0
	Dyspnea	0	0	0	0
	Sneezing	1	1	0	0
Eyes, nose	Conjunctivitis	1	1	0	0
	Rhinorrhea	1	1	0	0
Cardiovascular	Cardiovascular	0	0	0	0
Neurological	Headache	7	1	0	0
	Change in demeanor	1	1	0	0

Grade of reaction severity	# OIT doses	# Patients affected
Mild	638	14
Moderate	0	0
Severe	0	0

Adjustment to OIT daily dose	# OIT doses	# Patients affected
# Temporarily withheld doses	10	2
# Temporarily reduced	5	4
Permanently discontinued	1	1

Medications		# Doses medication administered	# Patients who received antihistamines
Antihistamines	Diphenhydramine	2	2
	Rupatadine	5	2
	Cetirizine hydrochloride	1	1
Epinephrine/Emergency room visit	Epinephrine	0	0
	Emergency visits or hospitalizations	0	0

^a^ 285 instances of mild recurrent abdominal pain occurred in one patient. 2 patients experienced < 30 instances of abdominal pain. The remaining 4 patients experienced < 5 instances of abdominal pain.

^b^ Self‐resolving cough without dyspnea or wheezing.

The most reported symptom during home‐dosing was 348 instances of abdominal pain, which was experienced by seven patients (Table 2). However, 285 instances of abdominal pain were attributed to one patient who had recurring daily mild gastrointestinal discomfort before withdrawal. Notably, this patient declined medication in all instances, and symptoms resolved without escalation. The second most frequently reported symptom was local oral discomfort, including 241 instances of lip, mouth, throat, or tongue itch, which affected 10 patients (Table 2).

Most home‐dosing‐related AEs occurred during the dose‐escalation phase. The median number of AEs per treatment day during the dose escalation period (daily 4–16 mg protein/nut) was 0.016 (IQR 0.003–0.054) for the 18 enrolled patients. Fourteen patients achieved the 30 mg protein/nut maintenance dosing. More than 50% of the patients experienced no AEs per treatment day during the maintenance period (IQR 0.000–0.003). Including the one patient who completed the exit OFC at 16 mg/nut, the overall median AEs per total number of treatment days during VLOIT was 0.014 (IQR 0.001–0.054).

Seven patients experienced 10 mild adverse reactions during dose‐escalation visits. The most common symptoms included local oral reactions (4 instances of itch, one instance of discomfort in the mouth) and four instances of sneezing (Supporting Information S1: Table S7). One patient received diphenhydramine for abdominal pain, which resolved without escalation.

#### 3.2.5 Very Low‐Dose Multi‐OIT Improves Parents' Perception of Social and Dietary Restrictions

To assess the impact of VLOIT on the lives of patients and families, quality of life was measured. Thirteen parents completed the Food Allergy Quality of Life Questionnaire ‐ Parent Form (FAQLQ‐PF) at baseline, post‐escalation, and the 18‐month exit visit. One child and two teenagers completed the respective child and teen FAQLQ forms for the three time points. The results from the two teenage participants showed a trend toward decrease in the scores for emotional Impact (EI), risk of accidental exposure (RAE), and allergen avoidance and dietary restrictions (AADR) categories of the survey (Supporting Information S1: Figure S1). The FAQLQ parent forms comparing baseline and 18‐month median scores of FAQLQ‐PF domains: EI, food‐related anxiety (FRA), and social and dietary restrictions (SDR) found SDR to improve significantly. The median score at baseline was 3 (IQR 2–5), which decreased to 1 (IQR 0.5–2.5) by the end of OIT (*p* < 0.01) (Supporting Information S1: Figure S2). EI and FRA scores were found to be non‐significant.

## 4 Discussion

Multi‐nut VLOIT is a novel approach that aims to explore very low‐dose multi‐nut oral immunotherapy, targeting a maintenance dose of only 30 mg/nut protein across 2–5 allergens in a pediatric population. Of the 18 enrolled participants, 14/18(78%) receiving the target maintenance dose of 30 mg per nut successfully consumed the maximum dose on the 18 months OFC or five times their baseline tolerated dose. Of these 14, 10/14 (71%) were able to consume the maximum dose of 2040 mg protein per nut.

Our findings suggest that a low and slow dose escalation with only three planned up‐dosing visits every 2 months may be beneficial for patients, demonstrating similar immunological outcomes to higher dose regimens. Notably, the median time in our study to reach 30 mg protein/nut was planned for approximately 27 weeks (6 months) but ultimately extended to 304.5 days (∼43 weeks), primarily due to COVID‐19 clinic restrictions. In comparison, Vickery et al. (2017), a 300‐ and 3000 mg peanut OIT trial required 42 weeks for dose escalation with 20 visits [10]. In the 2017 study, no significant differences were observed in the rates of IgE, IgG4, or SPT changes between the 300 and 3000 mg treatment groups. With our further 10‐fold reduction in target maintenance dose to 30 mg protein, suppression of IgE and log‐fold induction of IgG4 were demonstrated, using a similar treatment duration for dose escalation.

The time to reach the maintenance dose was extended due to COVID‐19. Thus, only one patient reached the maintenance dose at 6.5 months. There is a possibility that this delayed dose escalation may have impacted the OFC results as they had less time on the maintenance dose than planned. They may have benefited due to this low and slow approach. Sasamoto et al. (2021) reported three children with 75 mg walnut protein OIT [8]. One patient, who failed a 12‐month assessment for sustained unresponsiveness, continued 75 mg daily for 12 more months and achieved sustained unresponsiveness to 450 mg at 24 months [8] Itonaga et al. demonstrated that an extended “slow” approach may improve OFC outcomes in a stepwise wheat allergy protocol (52, 390, 1300, 5200 mg) [14]. In this investigation, participants testing positive while stepping up continued previous doses and reattempted after 6–12 months. Over 3 years, 70%, 58%, 53%, and 45% could consume 52 mg, 390 mg, 1300 mg, and 5200 mg, respectively [14].

Our results may be surprising given that OIT in older children and adults has demonstrated that the amount of food a participant can consume after OIT is proportional to the maintenance dose [15]. It is possible that by using 30 mg of protein from each nut, we benefited from some similarities in the nuts. For example, cashew and pistachio are highly cross‐reactive, as they share a strong phylogenetic relationship, which is reflected in the amino acid similarity and conserved three‐dimensional regions between their seed storage proteins [16]. On that account, children on both these nuts may be rather effectively getting a dose of 60 mg. However, even 60 mg is still very low dose and is a proof of concept that these doses can desensitize. We have an ongoing randomized controlled trial directly comparing only peanut OIT if 30 mg maintenance to 300 mg to avoidance which removes the possibility of cross desensitization [17].

Our study population had a low baseline threshold of reaction at only 10 mg protein/nut (IQR 4–144 mg), which may be another reason we had such significant threshold increases with only low doses. However, we limited to only children who could tolerate a 4 mg dose (6 mg cumulative) on the first desensitization day and all children with the highest entry OFC (reactive to a 300 mg protein dose) achieved non‐reactivity to the 1000 mg dose with only 30 mg dosing. Efficacy findings are in line with data from a recent sublingual immunotherapy trial, which applied a 10‐fold lower dose for peanut immunotherapy [18] and thus leads to consideration of how low can we go with OIT.

There have been concerns that a certain OIT maintenance dose is required to treat food allergy [19]. This present study continues to demonstrate that we have not yet identified the lower efficacy limit of OIT. To this point, one participant who had a baseline reactive dose of 10 mg and was treated with epinephrine achieved only 16 mg protein/allergen during the study period. And yet, at the 18‐month OFC, this participant was able to consume a cumulative tolerated dose of 440 mg protein/allergen with mild symptoms and displayed comparable changes in immunological parameters, including decreased SPT and blocking antibody induction compared to other participants. Importantly, there was no clinical or immunological evidence of increased allergen sensitization in participants. We did not see a reduction in tolerated food doses or a lack of IgG4 induction in any participant. We recognize IgG4 indicates exposure, not tolerance; however, many studies show a connection between IgG4 induction and reduced basophil degranulation as a biomarker of efficacy in allergen immunotherapy [20, 21, 22, 23]. Santos et al. demonstrated that peanut‐specific IgG4 prevents degranulation of peanut‐allergic basophils and mast cells and underlie the absence of clinical reactivity in peanut‐sensitized but tolerant children [24].

Our visits every 2‐month for dose escalation may improve the accessibility of OIT and reduce the burden on healthcare and patients. Rush oral immunotherapy has many adverse reactions. A long buildup phase for peanut OIT is safer and more tolerated [6, 25]. Palforzia, an FDA‐approved peanut OIT product, has standardized instructions for dose modification in the event of patients experiencing allergic reactions including “maintaining the dose level for longer than 2 weeks, reducing, or withholding.” [7, 26] Additionally, investigative studies of the efficacy and safety of Palforzia alternative dosing regimens from a 2‐year follow‐on study observed the highest desensitization rate in the group with the longest dosing duration (300 mg/day during 24–56 weeks) [27]. Our work suggests maintaining patients on a very low dose for an extended period may allow for patients, including those highly allergic patients who require a slower dose escalation schedule, to remain on OIT.

The entry OFC used a nut mix and not individual nut challenges. We cannot be certain that all were allergic to each treated nut within the context of individual OFCs. Strengths of this approach include our protocol, which is aimed at being practical, enabling the simultaneous treatment of all candidate tree nuts while reducing the patient burden of food challenges. By using a mix, dosing is limited by the most allergic nut and considers cross‐reactivity. Evidence of allergy was multifold: participants were referred by a community allergist, and inclusion criteria for nuts included a positive SPT to nut extract or fresh nut, positive sIgE to nut extract or component, prior clinical reactions, or failed food challenges to a nut.

Approximately 20 percent of the consented participants either did not meet the screening criteria of at least 4 mg or could not tolerate the initial desensitization. The threshold was set for inclusion because other OIT studies employed similar safety‐related requirements, and to demonstrate the effectiveness of the low‐dose multi‐OIT protocol across a broader food‐allergic population, rather than in those with very low thresholds. We believe this criterion should be revised in practice, recommending that these minimum thresholds be further lowered or eliminated to include these low‐threshold reactors. Lower start doses will be required to manage this low threshold population. As OIT is more widely adopted in the community, these measures will help improve accessibility in several ways.

Improvements in quality of life following low‐dose OIT were exploratory outcomes given the small sample size. Data collection was primarily conducted through FAQLQ parent surveys. Improvement in social and dietary restrictions (SDR) in the context of quality of life was expected, as low‐dose OIT is designed to be protective against accidental exposure. However, there were no significant improvements to emotional impact (EI) or food‐related anxiety (FRA) components of the FAQLQ. There may be a few underlying causes for these findings. Most notably, this study was conducted during the COVID‐19 pandemic, and parents were understandably anxious about accidental exposure and the use of emergency services. Also the FAQLQ was conducted on the 18 months visit prior to the results of the OFC, which may underlie parental anxiety. Potentially, it may be more important for parents and participants in a low‐dose protocol to observe changes in OFC threshold compared to those in high‐dose OIT (≥ 3000 mg protein). Visibly, 30 mg protein of nut butter, even in a 5x multi‐nut dose, would amount to less than a teaspoon or 3/5 of one peanut (250 mg protein). In contrast, 3000 mg of peanut protein is roughly equivalent to 12 peanuts. Considering larger sample sizes, some studies examining quality of life outcomes after OIT have found significantly improved parent outcomes following treatment [28]. Future investigations are needed to examine parent and participant perceptions and quality of life following very low‐dose OIT.

We did not have a placebo control group in the study, but results from placebo‐controlled OIT trials with comparable ages and timepoints suggest that the very low‐dose OIT protocol is preferable over placebo treatment of food allergies for improving OFC outcomes [10, 15, 29]. For example, Burks et al. egg‐OIT trial enrolled 55 children (ages 5–11) who received OIT (*n* = 40) or placebo (*n* = 15) [30]. At 10 months, 55% of the egg‐OIT group passed a 5g egg white OFC, while no placebo participants passed. At 22 months, 75% of the egg‐OIT group passed the 5g OFC, and placebo participants were not rechallenged due to prior performance [30].

Our safety data shows that most participants experienced AEs related to OIT, but all were mild, and few adjustments to daily dosing and/or antihistamines were required. No AEs associated with OIT were graded as severe, and no participants required epinephrine or hospitalization. Participants were still provided with all safety advice following dosing, including instructions to avoid strenuous exercise. A limitation of this study is that we did not explore whether lifestyle restrictions are necessary with very low dosing.

Other limitations include the open‐label OFCs and dosing, small sample size, and the absence of a placebo group. The main goal was to evaluate if there was a signal of efficacy. The COVID‐19 pandemic caused scheduling difficulties. Participants had less time on maintenance than planned whereas the final OFC was kept close to 18 months which may have under‐estimated efficacy of our regimen. Furthermore, we used mixed‐nut OFCs and thus changes in individual allergen thresholds were not assessed.

The doses used in Low‐dose multi‐OIT may be compared and contrasted with those used in other approaches, such as with food ladders, epicutaneous, and sublingual immunotherapy. No direct comparisons exist, but several features, including the flexibility of ladders and the extended low dosing in epicutaneous or sublingual therapies, support the use of lower and slower doses [31]. Food ladders gradually reintroduce allergenic foods, starting with heated foods, using 0.1–0.3 g of milk or egg, then escalating to 2–8 g of heated protein. They are less formal but increasingly used to reduce dietary restrictions and for potential therapeutic effects for cow's milk‐ and egg‐allergic children, often at home with moderate monitoring [32, 33]. In contrast, sublingual immunotherapy (1–4 mg) and epicutaneous methods like the Viaskin patch (250 μg) require more clinic visits and may suit those reacting to lower thresholds [18, 34]. These low doses are effective; for example, in a recent 4‐mg peanut SLIT trial, the mean tolerated dose increased from 48 to 2723 mg over 48 months, with 36% reaching 5000 mg SCD [18].

Notable strengths of the study are the inclusion of baseline and post‐treatment OFCs and immunological evaluations. Patients who encountered difficulties with escalating doses were permitted to continue their treatment. This study successfully illustrates the adaptability of achievable target OIT dosing and its advantages for patients who present with multiple allergies and may benefit from flexible dosing protocols.

In conclusion, 18 months of multi‐nut VLOIT at only 30 mg of protein per nut met multiple outcomes for desensitization and had no treatment‐related anaphylactic events. Of the 14 participants treated per protocol, 13/14 (93%) were able to consume at least the 300 mg dose and at least five times their baseline amount and 10 could consume our maximal challenge of cumulative 2040 mg per nut. We demonstrate this low maintenance dose provides expected protection against accidental exposure and effectively induces immunoregulatory mechanisms. A low‐dose protocol can be paired with dose‐escalation visits every 2 months (or even less frequently), resulting in fewer clinic visits and a reduced burden on families and healthcare providers.

## Author Contributions

**Julia E. M. Upton:** conceptualization, investigation, funding acquisition, methodology, supervision, writing – review and editing, formal analysis, writing – original draft, resources, data curation, project administration, visualization, validation. **Carmen H. Li:** writing – original draft, methodology, writing – review and editing, data curation, visualization. **Alireza Berenjy:** data curation, writing – review and editing, project administration. **Alana Galper:** writing – review and editing, data curation, project administration. **Xiaojun Yin:** methodology, writing – review and editing, formal analysis, project administration. **Alper Celik:** formal analysis, software, writing – review and editing. **Lucy Duan:** writing – review and editing, project administration. **Samantha Wong:** writing – review and editing, project administration, methodology. **Christina M. Ditlof:** writing – review and editing, project administration. **Kristen E. San Diego:** writing – review and editing, methodology, data curation. **Jennifer A. Hoang:** writing – review and editing, project administration, data curation. **Moshe Ben‐Shoshan:** writing – review and editing. **Akash Kothari:** writing – review and editing. **Lisa Hung:** writing – review and editing. **Mikhail Monteiro:** writing – review and editing, formal analysis. **Wut Hmone Phue:** writing – review and editing. **Theo J. Moraes:** writing – review and editing. **Thomas Eiwegger:** writing – review and editing, conceptualization, methodology, supervision, data curation, formal analysis, project administration, writing ‐ original draft, validation, visualization, resources, investigation.

## Funding

This work was supported by The Hospital for Sick Children (SickKids Food Allergy and Anaphylaxis Program, start‐up funds from the SickKids Research Institute and Department of Pediatrics, and the Restracomp Graduate Scholarship awarded to C.H.L., L.H., and A.K.). C.H.L. is a recipient of the Canadian Institutes of Health Research (CIHR) Frederick Banting and Charles Best Canada Graduate Scholarship.

## Conflicts of Interest

T.E. reports to act as local P.I. for company sponsored trials by D.B.V. Therapeutics, Greer Stallergens, and sub‐investigator for Regeneron and ALK‐Abelló. He is Co‐Investigator or scientific lead in three investigator‐initiated oral immunotherapy trials supported by the SickKids Food Allergy and Anaphylaxis Program and serves as an associate editor for Allergy. He/his lab received unconditional/in‐kind contributions from Macro Array Diagnostics and an unrestricted grant from ALK‐Abelló. He holds advisory board roles for ALK‐Abelló, VAMED, Nutricia/Danone and Aimmune. TE reports lecture fees from Novartis, ThermoFisher, Nutricia/Danone, Aimmune, ALK‐Abelló. JEMU reports grants and personal fees from ALK‐Abelló, D.B.V. Technologies, personal fees from Bausch Health, Pfizer, Viatris, Pharming Group N.V., donation of drug for a trial from Novartis, grants from Food Allergy Anaphylaxis Program (SickKids), CIHR, Board of Director at Canadian Society of Allergy and Clinical Immunology; Healthcare Advisory Board Food Allergy Canada. MBS reports personal fees from ALK‐Abelló, D.B.V. Technologies, personal fees from Bausch Health, Pfizer, Viatris, P.I. for studies with Sanofi Novartis and ALK, grants from Montreal Children's Foundation, CIHR, Board of Director at Canadian Society of Allergy and Clinical Immunology; Healthcare Advisory Board Food Allergy Canada. All other authors report no conflict of interest.

## Supporting information


Supporting Information S1

## Data Availability

Research data are not shared.
