# Supplementary material for: Safety and Efficacy of Very Low‐Dose Multi‐Nut Oral Immunotherapy in Children
Source: Clin Transl Allergy. 2025 Dec 12;15(12):e70125. doi: 10.1002/clt2.70125 (PMC12700494; doi:10.1002/clt2.70125)
Supplement: Supplementary file 1 — Supporting Information S1 [file CLT2-15-e70125-s001.docx]

**Supplementary: Safety and efficacy of Very Low-Dose Multi-Nut Oral Immunotherapy in Children**

**Authors:** Julia E.M. Upton MD MPH^1,2*^, Carmen H. Li MSc^3,4*^, Alireza Berenjy MD^3^, Alana Galper MPH RD^3^, Xiaojun Yin PhD^3^, Alper Celik PhD^5^, Lucy Duan MD^2^, Samantha Wong RD^3^, Christina M. Ditlof MSc^3,6^, Kristen E. San Diego BSc^3^, Jennifer A. Hoang MSc MDSc^3,6^, Moshe Ben-shoshan MD^7^, Akash Kothari MSc^3,4^, Lisa Hung PhD^3,6^, Mikhail Monteiro BSc^3^, Wut Hmone Phue PhD^3^,Theo J. Moraes MD^2,3^ Thomas Eiwegger MD^3,6,8,9^

*Co-first Author

(1) Division of Immunology and Allergy, Food Allergy and Anaphylaxis Program, The Hospital for Sick Children

(2) Department of Pediatrics, University of Toronto, Toronto, ON, Canada

(3) Translational Medicine Program, Research Institute, Hospital for Sick Children, Toronto, ON, Canada

(4) Institute of Medical Science, University of Toronto, Toronto, ON, Canada,

(5) Centre for Computational Medicine, Research Institute, Hospital for Sick Children, Toronto, ON, Canada

(6) Department of Immunology, University of Toronto, Toronto, ON, Canada

(7) Division of Allergy and Clinical Immunology, Department of Pediatrics, Montreal Children's Hospital, Montreal, Quebec, Canada.

(8) Karl Landsteiner University of Health Sciences, Krems, Austria

(9) Department of Pediatric and Adolescent Medicine, University Hospital St. Pölten, St. Pölten, Austria

**Running Title: Very Low-Dose Multi-Nut OIT**

**Correspondence:**

**Thomas Eiwegger, MD**

Thomas Eiwegger, Department of Pediatric and Adolescent
Medicine, University Hospital St. Pölten, Dunant-Platz 1,
3100 St. Pölten, Austria.
Email: [thomas.eiwegger@kl.ac.at](mailto:thomas.eiwegger@kl.ac.at)

### Study power calculation:

For the primary outcome of efficacy, in this very well-defined cohort we expect less than 20% will not meet the inclusion criteria by reacting to 4mg protein/nut and none will be not allergic to their nut mix.^1,2^ Therefore, we will challenge 18 patients to enroll 15. Anticipated efficacy is that the OIT will increase the reaction threshold. At an Alpha 0.05 and Beta 0.2 with a Power 0.8. We anticipate 60% will have at least a 5X increase in threshold, versus no more than 20% historically. At an Alpha 0.05 and Beta 0.2 with a Power 0.8, 9 patients will show this difference (60% vs 20%) with a one sample proportion test.

### Skin prick test:

Skin prick tests were performed with peanut and nut extracts from Omega Pharmaceuticals (Canada) and with Medipoint (United States) steep lancets used in the Hospital for Sick Children Allergy Clinic. Saline was used as the negative control and histamine extract as the positive control.

### Blood sample collection:

A blood sample for basophil activation testing (15ml in an ethylenediaminetetraacetic acid tube sent immediately to the Eiwegger lab) and for specific IgE and IgG4 testing (4 ml serum sample stored at the respective site at -20C) will be drawn by the research nurse or a trained person with phlebotomy privileges. The blood sample was labelled with the study participant’s ID number, date and time when sample was collected.

### Component resolved allergen-specific IgE and IgG4 measurements:

Allergen-specific IgE was measured with the Allergy Xplorer (ALEX^2^) array kit, an ELISA-based quantitative *in vitro* colorimetric multiplex assay (Macro Array Diagnostics, Austria) according to manufacturer’s instructions as demonstrated in prior publications. ^3,4^ For IgE quantification, patient serum was diluted 1:5 and incubated on the ALEX^2^ array chip containing 117 extracts and 178 molecular allergens. Positive sensitizations were defined as ≥0.30 kU/L.

For IgG4 quantification, patient serum was diluted, based on needs and timepoint of sampling 1:10, 1:100, 1:1000, and 1:10000 and incubated on individual ALEX^2^ arrays to accommodate several log-fold increases in IgG4 during OIT. The lowest serum dilution reading <27.5 kU/L in the dynamic working range of the ALEX^2^ for each specific allergen extract/component at each timepoint was selected and corrected for dilution factor and assigned arbitrary units (AU).^3,4^ The secondary detection antibody for IgG4 evaluation (mouse IgG1κ anti-human IgG4, Macro Array Diagnostics, Austria) was provided by the manufacturer.

**Table 1S. Demographics of participants who did not meet screening criteria of tolerating 4mg protein/nut.**

| **Participant**  **(N=4)** | **1** | **2** | **3** | **4** |
| --- | --- | --- | --- | --- |
| **Sex** | M | M | M | M |
| **Age at Start of OIT (Years)** | 2 | 4 | 14 | 12 |
| **Atopic History (0 No, + Yes)** |  |  |  |  |
| **Asthma** | 0 | 0 | 0 | + |
| **Atopic Dermatitis** | 0 | 0 | 0 | 0 |
| **Allergic Rhinitis** | 0 | + | 0 | + |
| **Food Allergy** |  |  |  |  |
| **Walnut** | + | + | + | + |
| **Pistachio** | 0 | + | + | + |
| **Cashew** | + | 0 | + | + |
| **Hazelnut** | + | + | + | + |
| **Peanut** | + | + | 0 | + |
| **Macadamia** | 0 | 0 | + | 0 |
| **Almond** | + | + | 0 | 0 |
| **Baseline Oral Food Challenge** |  |  |  |  |
| **Cumulative Successfully Consumed Dose (mg protein/nut)** | 0 | 0 | 1 | 1 |
| **Eliciting Dose per Allergen (mg protein /nut)** | 1 | 1 | 4 | 4 |

**Table 2S. Study visits for Very Low-Dose Multi-Oral Immunotherapy.**

|  | Study Eligibility and oral food challenge | Initial Desensitization | Dose Escalation (over 6 months), visits every 2 months | | | Maintenance (over 1 year),  visits every months | | | Final Challenge |
| --- | --- | --- | --- | --- | --- | --- | --- | --- | --- |
| Clinic Visit # | 1 | 2 | 3 | 4 | 5 | 6 | 7 | 8 | 9 |
| Timeline* |  |  | 2 months post visit 2 | 4 months post visit 2 | 6 months post visit 2 | 3 months post visit 5 | 6 months post visit 5 | 9 months post visit 5 | 12 months post visit 5 |
| Approx.  Cumulative time (weeks) | 0 | 2 | 10 | 19 | 25 | 38 | 50 | 62 | 75 |
| Oral Food Challenge | X |  |  |  |  |  |  |  | X |
| Nut Dose Consumed |  | X | X | X | X |  |  |  |  |
| Skin Prick Test | X |  |  |  | X |  |  |  | X |
| Blood Draw | X |  |  |  | X |  |  |  | X |
| Intravenous Line** | X |  |  |  |  |  |  |  | X |
| Physical Exam | X |  |  |  |  |  |  |  |  |
| Limited physical exam (skin, chest, vital signs) |  | X | X | X | X | X | X | X | X |
| QOL Survey | X |  |  |  | X |  |  |  | X |
| Diary Review |  |  | X | X | X | X | X | X | X |

*Note that dose escalations are personalized, and timeline can be extended if dose escalations take longer, a month has been approximated at 4-5 weeks

**Intravenous line insertion is at the discretion of the study doctor

OFC: Oral food challenge, QOL: Food allergy quality of life survey, SPT: Skin prick test

**Table 3S. Oral food challenge doses**

| OFC#1: Oral Food Challenge to multi-nuts (up to 5) at Baseline | | |
| --- | --- | --- |
| Time (minimum time between doses is 15 min) | Dose (of each nut protein, in a mix) | Cumulative Dose |
| 0 |  |  |
| 15 | 1 mg | 1 mg |
| 30 | 3 mg | 4 mg |
| 45 | 10 mg | 14 mg |
| 60 | 30 mg | 44 mg |
| 75 | 100 mg | 144 mg |
| 90 | 300 mg | 444 mg |
| OFC#2: Oral Food Challenge to multi-nuts (up to 5) following OIT | | |
| Time (minimum time between doses is 15 min) | Dose (of each nut protein, in a mix) | Cumulative Dose |
| 0 | 10 mg | 10 mg |
| 15 | 30 mg | 40 mg |
| 30 | 100 mg | 140 mg |
| 45 | 300 mg | 440 mg |
| 60 | 600 mg | 1040 mg |
| 75 | 1000 mg | 2040 mg |

**Table 4S. Dose escalation steps**

| Dose Escalation # | Time +/- 2 weeks | Approx. each nut protein (mg) | Approx. increase percentage dose | Measured amount of each nut butter (teaspoon)  ~3000 mg of protein in 1 tablespoon (3 teaspoons) of nut butter |
| --- | --- | --- | --- | --- |
| Introduce multi-OIT | 0 | 2.0 mg initially followed by 4.0 mg 1 hour later | n/a | 2.0 mg protein weighed at the research center  Approx. 4.0 mg is 1/64 diluted ¼ in oil or water, use “drop spoon” |
| Updose 1 | 8 weeks | 7.81 mg | 100 | 1/64 diluted in 1/2 oil or water, use “drop spoon” |
| Updose 2 | 16 weeks | 15.63 mg | 100 | 1/64, use “drop spoon” |
| Updose 3 | 24 weeks | 31.25 mg | 100 | 1/32 teaspoon “1/32 =Smidgen” |
| Maintenance | to 84 weeks | | | |

**Table 5S. OIT Participant Baseline Characteristics**

| **OIT Treatment Nut** | **# OIT Participants (n=18)** | **Positive SPT to Nut Extract**  **(>3 mm)** | **Positive IgE >0.35 KU_A_/L Allergen Components** | **Positive IgE >0.35 KU_A_/L**  **2S Albumin Seed Storage Protein** | **Clinical History to Allergen** | **Positive Basophil Activation Test** |
| --- | --- | --- | --- | --- | --- | --- |
| **Walnut** | 12 | 11* (n=12) | 12 (n=12) | 11 (n=12) Jug r 1 | 6 (n=6) | 12 (n=12) |
| **Pistachio** | 14 | 13** (n=14) | 13** (n=14) | 13 (n=14) Pis v 1 | 4 (n=4) | 11** (n=12) |
| **Cashew** | 14 | 14 (n=14) | 13 **(n=14) | 12 (n=14) Ana o 3 | 9 (n=9) | 11** (n=12) |
| **Hazelnut** | 9 | 8(†) (n=9) | 7 (n=9) | 6 (n=9) Cor a 14 | 3 (n=3) | 7 (n=7) |
| **Peanut** | 11 | 11 (n=11) | 11 (n=11) | 11 (n=11) Ara h 2 | 8 (n=8) | 10 (n=10) |
| **Macadamia** | 1(‡) | 0 (n=1) | 0 (n=1) | 0 (n=1) Mac i | 1 (n=1) | Not Tested |

(n) = Number of participants with available data.

(*) Participant with a negative walnut SPT had failed an open walnut challenge 1 month prior to screening and had a positive pecan SPT.

(**) Participant with a negative pistachio extract SPT had a positive test to fresh pistachio and cashew extract. The participant had failed a pistachio challenge 4 months prior to screening. The same participant had a low IgE components and BAT responses to pistachio and cashew. This participant received cashew, pistachio, peanut OIT.

(†) Participant with a negative hazelnut SPT had positive sIgE to Cor a 14 (2.25 KU_A_/L) and had a history of reacting to walnut after a negative SPT.

(‡) Participant may possibly be non-allergic to macadamia. The participant met criteria for study based on pistachio and cashew. Macadamia was included based on family request.

Non-responder patients were not included in BAT results.

**Table 6S. OFC Reaction Scoring**

| **Participants**  **N=18** | **COFAR -OFC1** | **COFAR -OFC 2** |
| --- | --- | --- |
| 1 | COFAR 3 | COFAR 0 |
| 2 | COFAR 2 | Participant Withdrawn |
| 3 | COFAR 2 | COFAR 3 |
| 4 | COFAR 2 | COFAR 0 |
| 5 | COFAR 3 | COFAR 0 |
| 6 | COFAR 3 | COFAR 0 |
| 7 | COFAR 2 | COFAR 0 |
| 8 | COFAR 3 | COFAR 0 |
| 9 | COFAR 3 | Participant Withdrawn |
| 10 | COFAR 1 | COFAR 0 |
| 11 | COFAR 2 | COFAR 2 |
| 12 | COFAR 2 | COFAR 3 |
| 13 | COFAR 2 | COFAR 3 |
| 14 | COFAR 1 | COFAR 0 |
| 15 | COFAR 2 | COFAR 3 |
| 16 | COFAR 2 | COFAR 0 |
| 17 | COFAR 3 | Participant Withdrawn |
| 18 | COFAR 2 | COFAR 0 |
| comparison | 2 (IQR 1,3) | 0 (IQR 0, 2)*** |

***Wilcoxon, p-value: 0.0078

**Table 7S. Adverse Events During Dose Escalation Visits**

| **Dose Escalation Visit Associated Adverse Events during Very Low-Dose Multi-OIT** | | | |
| --- | --- | --- | --- |
| **Total Number of AEs occurring during Dose Escalation Visits** | | **# Patients with Dose Escalation AEs**  **/ # Enrolled Patients** | |
| 10 | | 7/18 | |
| **Symptom Categories** | | **# Instances of symptoms during Dose Escalation Visit** | **# Patients affected** |
| **Dermatological** | **Itch** | 1 | 1 |
|  | **Eczema flare** | 0 | 0 |
|  | **Hives, Urticaria, Rash** | 1 | 1 |
| **Local Oral Reaction** | **Itch of lips, mouth, throat, tongue** | 4 | 4 |
|  | **Hive on lips, mouth** | 0 | 0 |
|  | **Redness, swelling of lips, mouth** | 0 | 0 |
|  | **Pain or discomfort of lips, mouth, throat, tongue** | 1 | 1 |
| **Gastrointestinal** | **Nausea** | 0 | 0 |
|  | **Vomiting** | 0 | 0 |
|  | **Diarrhea** | 0 | 0 |
|  | **Abdominal pain** | 2 | 2 |
|  | **Acid reflux** | 0 | 0 |
| **Respiratory** | **Cough w/o dyspnea**** | 0 | 0 |
|  | **Sneezing** | 4 | 4 |
| **Eyes, Nose** | **Conjunctivitis** | 0 | 0 |
|  | **Rhinorrhea** | 0 | 0 |
| **Cardiovascular** | **Cardiovascular** | 0 | 0 |
| **Neurological** | **Headache** | 0 | 0 |
|  | **Change in demeanor** | 0 | 0 |
| **Grade of Reaction Severity** | | | **# Patients affected** |
| **Mild** | | 9 | 7 |
| **Moderate** | | 0 | 0 |
| **Severe** | | 0 | 0 |
| **Adjustment to OIT Daily Dose following Dose Escalation Visit** | | | **# Patients affected** |
| **# Temporarily reduced daily dosing amount** | | 2 | 2 |
| **Permanently discontinued** | | 0 | 0 |
| **Medications** | | **# Doses Medication Administered** | **# Patients affected** |
| **Antihistamines** | **Diphenhydramine** | 1 | 1 |
|  | **Rupatadine** | 0 | 0 |
|  | **Cetirizine hydrochloride** | 0 | 0 |
| **Epinephrine/**  **Emergency Room Visit** | **Epinephrine** | 0 | 0 |
|  | **Emergency visits or hospitalizations** | 0 | 0 |

**Self-resolving cough without dyspnea or wheezing


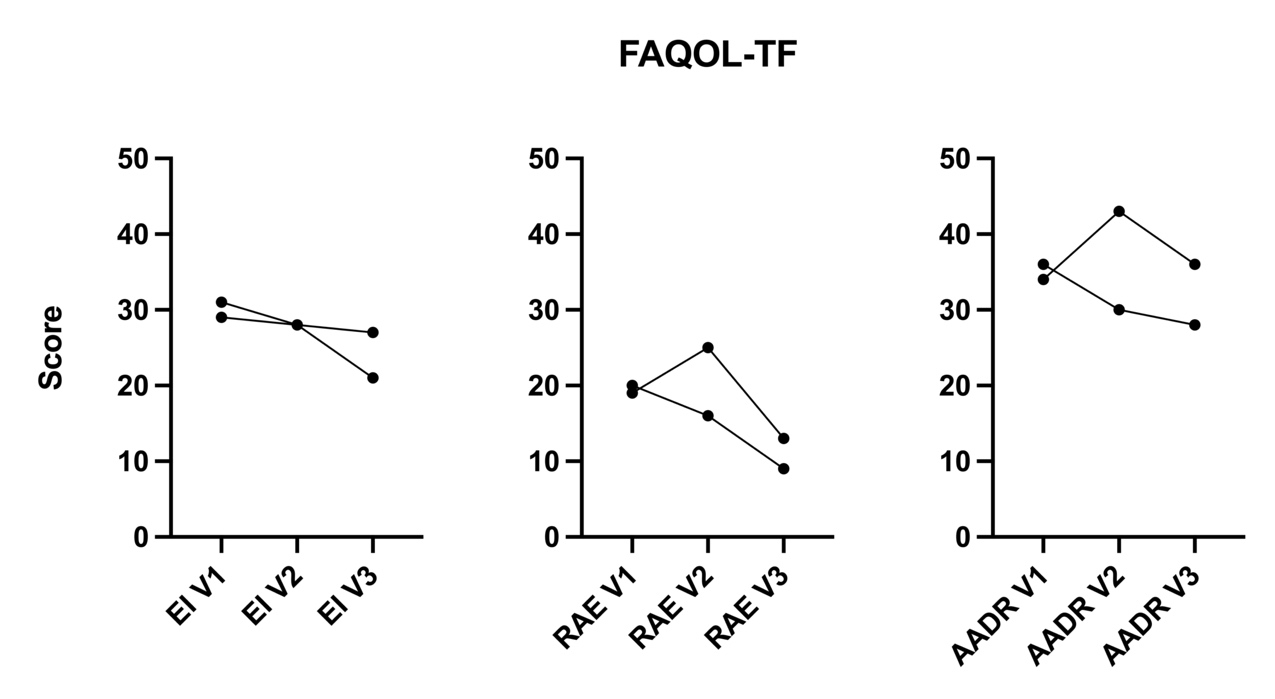


**Figure S1. Food allergy quality of life questionnaire teen form.**

Questionnaires were conducted at baseline (V1), post-escalation visit (reach 30mg) (V2), and end of maintenance exit visit at 18 months (V3). Individual scores (N=2) for the three subsections were plotted: emotional impact (EI), risk of accidental exposure (RAE), and allergen avoidance and dietary restrictions (AADR).


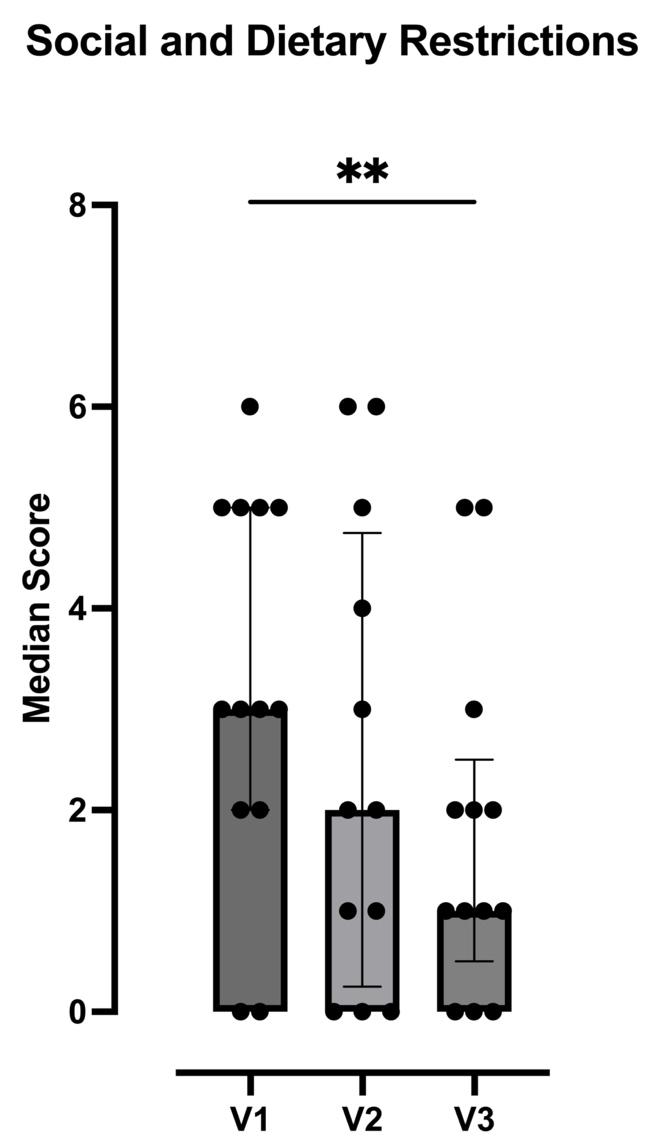


**Figure S2. Food allergy quality of life questionnaire parent form social and dietary restriction score.**

The median scores are plotted with the interquartile range. Questionnaires were conducted at baseline (V1), post-dose escalation visit (V2), and end of maintenance exit visit at 18 months (V3). 13 parents completed the food allergy quality of life questionnaire. Bars are plotted median and IQR. Friedman test and Dunn’s multiple comparison (N=12, p<0.05, Dunn’s test V1 vs. V3 p<0.05). Wilcoxon signed the ranks test (N=13, V1 vs. V3 p<0.01). **(p<0.01)

**Supplementary References**

1. Houben GF, Baumert JL, Blom WM, Kruizinga AG, Meima MY, Remington BC, et al. Full range of population Eliciting Dose values for 14 priority allergenic foods and recommendations for use in risk characterization. Food Chem Toxicol 2020; 146:111831.
2. PALFORZIA [^8^](#_ENREF_8). Brisbane, CA: Aimmune Therapeutics, Inc.
3. Duan L, Celik A, Hoang JA, Schmidthaler K, So D, Yin X, et al. Basophil activation test shows high accuracy in the diagnosis of peanut and tree nut allergy: The Markers of Nut Allergy Study. Allergy 2021; 76:1800-12.
4. Duan L, Hoang JA, Kothari A, Eiwegger T, Vadas P. Shellfish allergy is a risk factor for cricket anaphylaxis. J Allergy Clin Immunol Pract 2020; 8:2396-8.e1.
5. Brown SG. Clinical features and severity grading of anaphylaxis. J Allergy Clin Immunol 2004; 114:371-6.
